# Using Historic and Contemporary Genomes to Assess the Genetic Consequences of a Population Decline in an Endangered Tern Population

**DOI:** 10.1111/eva.70192

**Published:** 2026-01-02

**Authors:** Anna Schnelle, Robert E. Rollins, Ingo A. Müller, Martin Irestedt, Jacopo G. Cecere, Lorenzo Serra, Jorge S. Gutiérrez, Jose A. Masero, Markus Risch, Sandra Bouwhuis, Miriam Liedvogel

**Affiliations:** ^1^ Institute of Avian Research “Vogelwarte Helgoland” Wilhelmshaven Germany; ^2^ Department of Bioinformatics and Genetics Swedish Museum of Natural History Stockholm Sweden; ^3^ Department of Zoology Stockholm University Stockholm Sweden; ^4^ Area Avifauna Migratrice, Istituto Superiore Per la Protezione e la Ricerca Ambientale (ISPRA) Ozzano dell'Emilia Italy; ^5^ Department of Anatomy, Cell Biology & Zoology Faculty of Science, University of Extremadura Badajoz Spain; ^6^ Bündnis Naturschutz in Dithmarschen e.V. Hemmingstedt Germany; ^7^ Department of Biology and Environmental Sciences Carl von Ossietzky University of Oldenburg Oldenburg Germany

**Keywords:** conservation project, endangered population, historic DNA, population genetics

## Abstract

Many migratory species have experienced severe population declines, but the genetic consequences of such declines are still rarely assessed. The last Central European population of gull‐billed terns ( *Gelochelidon nilotica* ) has declined from 500 breeding pairs in the 1940s to 52 in 2025, whereas Mediterranean populations of this migratory waterbird still thrive. Here, we compare whole‐genome sequencing (WGS) data among the declining population, two thriving populations and the ancestors of the declining population. We find comparable nucleotide diversity, but lower observed heterozygosity in the Central European population compared to the Mediterranean populations. The contemporary samples show some population structure as well, although admixture analyses and low genetic differentiation (*F*_ST_) still suggest potential population connectivity. Museum specimens from the historic population reveal an increased level of genetic diversity compared to the contemporary population, with effective population size estimates suggesting two past population declines. While inbreeding coefficients (*F*_ROH_) in the current Central European population are significantly higher than in the historic population, they are similar to those in the Mediterranean populations. These results suggest that population structure may be emerging, and that although inbreeding is not yet at worrisome levels in the last Central European population of gull‐billed terns, it may be on the rise. If this endangered population remains small and isolation manifests, the effects of inbreeding depression may become more pronounced over time, potentially reducing fitness and increasing the risk of extinction.

## 1 Introduction

Concurrent with population declines, species experience reductions in their genetic diversity compared to historic levels (Frankham et al. 2017), with an estimated global loss within populations of vertebrates of about 6% since the Industrial Revolution (Leigh et al. 2019). Factors such as range contractions and increasing isolation between populations, for example due to habitat degradation, can restrict gene flow (Athrey et al. 2012). For small isolated populations, such reduced or arrested exchange of genetic material can elevate both the risk and the rate of extinction (Markert et al. 2010; Crooks et al. 2017; Frankham et al. 2017). Genomic erosion, for example, may lead to an inability of populations to adapt (Diamond and Martin 2020; Bosse and van Loon 2022) if the standing genetic variation is too small to keep pace with the rapid environmental changes many populations currently face (Willi et al. 2006).

Conservation efforts may be optimized when the effects of population declines on genetic structure and diversity are known and can be taken into account (Pierson et al. 2016; Frankham et al. 2017; Cavill et al. 2024; Smart et al. 2025). Such knowledge can be gained by adopting comparative genomics approaches across spatial and temporal scales. Spatial comparisons allow assessment of the contemporary genetic connectivity among populations, and incorporating temporal perspectives in genetic studies has become increasingly popular in recent years (Habel et al. 2014; Jensen and Leigh 2022; Clark et al. 2023; Cavill et al. 2024), given that examining genetic processes at only one single point in time often provides limited information about historic processes (Husemann et al. 2012; Díez‐del‐Molino et al. 2018; Jensen and Leigh 2022). In the latter case, the analysis of museum specimens can offer important insights into temporal changes in gene flow and population structure, as well as the adaptive potential of species, enabling assessments of how populations have responded to change (Ramírez et al. 2013; Benham and Bowie 2023).

The gull‐billed tern ( *Gelochelidon nilotica* ) is a long‐lived and globally distributed tern species with highly fragmented breeding areas (BirdLife International 2019). In Europe, the species' breeding range has declined over the last century (Møller 1975a, 1975b; Sánchez et al. 2004), with extant populations primarily concentrated in Mediterranean and Eastern European countries. Historically, however, gull‐billed terns are known to have bred across Central Europe. Up to 200 breeding pairs, for example, occupied the Alpine foothills before river canalization led to their extinction around 1930 (Berndt 2018). Similarly, a Danish population dramatically declined from c. 500 breeding pairs in the 1940s to 52 in 2025 as habitat destruction (Møller 1975a) resulted in a shift of the breeding area towards Germany (Berndt 2018; Risch et al. 2018). This German population is now the last Central European population, listed as critically endangered on the national level, and internationally protected by the African‐Eurasian Migratory Waterbird Agreement (AEWA) and the EU Birds Directive. Although the population retains genetic variation on the mitogenome (Schnelle, Rollins, et al. 2024), the extent of variation on the nuclear genome, as well as the genetic impact of the population decline and historic genetic diversity levels, have so far remained unexplored.

Here, we therefore compare genetic diversity indices and assess population genetic structure across three extant European gull‐billed tern populations using whole genome sequencing (WGS) data, thereby elevating the genetic information on contemporary European populations of gull‐billed terns and assessing whether the patterns observed using mitochondrial DNA (mtDNA) are also reflected in the nuclear genome. This is important because mtDNA is only maternally inherited, has a relatively fast evolutionary rate, and only represents a single non‐recombining locus, such that it may not allow us to fully capture all genetic processes. In addition to using contemporary genomes from the endangered German, as well as two thriving Spanish and Italian populations, we also include historic genetic data to assess the putative impact of the drastic population decline and estimate the long‐term effective population size (*N*_e_) to reveal potential impacts of genetic drift. To do so, we benefit from museum specimens collected between 1900 and 1928 that represent members of the pre‐decline population. Overall, we aim to conduct the first genome‐wide characterization of European gull‐billed tern populations and improve our understanding of their genetic connectivity in order to inform conservation strategies for the endangered last Central European population.

## 2 Methods

### 2.1 Sample Collection and DNA Extraction

**FIGURE 1:**
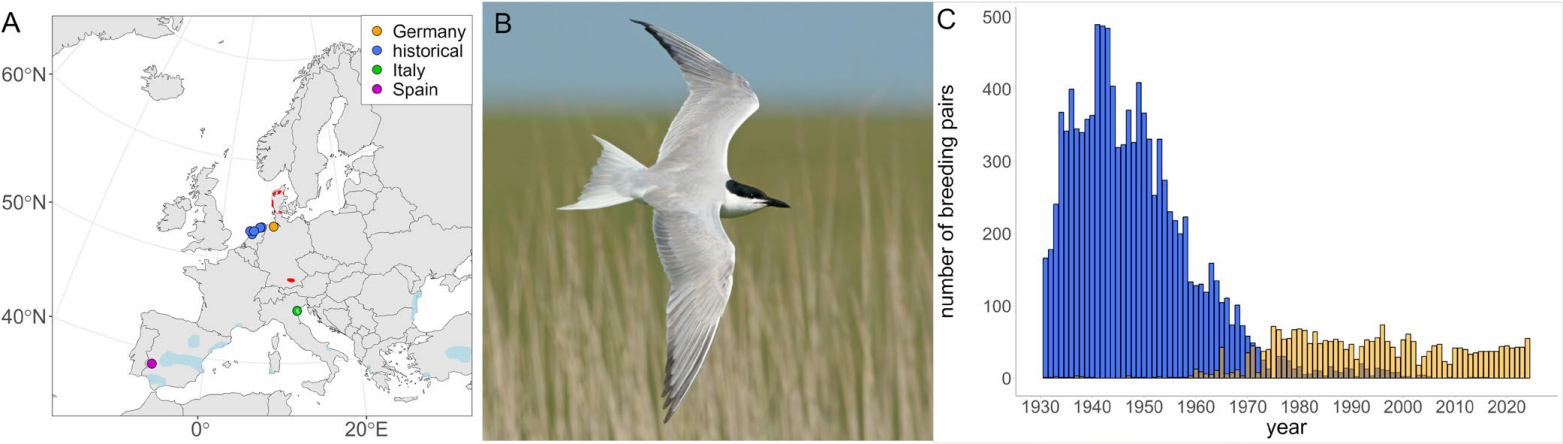
(A) European breeding populations of gull‐billed terns with the historic distribution (in red), the current distribution (in light blue) and the sample locations (in color coded circles: Germany in orange, Italy in green, Spain in purple; historic in dark blue). (B) Picture of a gull‐billed tern. (C) Population trend for the former Danish (dark blue) and current German (orange) population of gull‐billed terns (Møller 1975a; Berndt 2018).

To quantify contemporary genetic variation in European gull‐billed terns, we created a dataset consisting of 29 previously sampled birds (Schnelle, Rollins, et al. 2024), of which 17 belonged to the endangered German population and six to each of a thriving Italian and Spanish population (Figure 1A,B). Sampling of the German birds was approved by the state government of Schleswig‐Holstein (license V242–19875/2021 (30–4/21)). Capture, handling and sample collection in Italy were carried out by the Italian Institute for Environmental Protection and Research (ISPRA) at the Adriatic population (Scridel et al. 2023), under the authorization of Law 157/1992 [Art. 4(1) and Art. 7(5)], which regulates research on wild birds in Italy. The Spanish samples originated from the Extremadura population (Villegas et al. 2013), where sampling was approved by the Ethics Committee of the University of Extremadura (license 112/2020) and the Government of Extremadura (license CN0001/23/ACA). Unlike the German population (Figure 1C), the Italian and Spanish populations have not undergone a drastic decline in recent decades.

Genomic DNA of contemporary samples was extracted following a modified salt extraction protocol (Aljanabi and Martinez 1997). The concentration of the extracted DNA was measured on a Nanodrop spectrophotometer (Thermo Fisher Scientific) before it was sent to a commercial company (Novogene, Munich, Germany) for library preparation and whole genome sequencing. Sequencing was performed on an Illumina NovaSeq machine, producing paired‐end reads of 150 bp.

In addition to blood samples from the contemporary populations, we acquired 20 toe pads from museum specimens (14 adults, six fledglings), which were collected between 1900 and 1928 during migration through the Netherlands (Figure 1A). These samples were provided by the Naturalis Museum of Biodiversity in Leiden, the Netherlands. As only the former Danish population of gull‐billed terns migrated through the Netherlands during that time (Gloe and Møller 1978), these specimens can be considered ancestors of the extant German population. For extraction and library preparation of historic DNA (hDNA) we followed the procedures described by Irestedt et al. (2022). In brief, genomic DNA was extracted using the DNeasy Blood and Tissue kit (Qiagen, Hilden, Germany), and libraries were prepared following the protocol by Meyer and Kircher (2010) with modifications tailored for avian museum samples (Irestedt et al. 2022). These modifications included, for example, USER enzyme (New England Biolabs) treatment during the initial library preparation step to remove uracil residues, thereby mitigating the characteristic deamination patterns of hDNA (Briggs et al. 2010; Irestedt et al. 2022). Following indexing (four dual‐indexed libraries per individual), libraries from five to six individuals were pooled per sequencing lane. Sequencing was performed on the Illumina NovaSeq × Plus platform using the 10B flowcell at 300 cycles, resulting in 2 × 150 bp reads. Whole‐genome Illumina sequencing of the historic samples was conducted at the National Genomics Infrastructure (NGI) in Stockholm.

### 2.2 Read Processing and Genome Assembly

Individual raw sequence reads were processed using a custom variant calling pipeline (Weissensteiner et al. 2025). First, adapter trimming and quality control of raw fastq files (historic and contemporary) were performed using *bbduk* (v. 38.90, https://sourceforge.net/projects/bbmap/) with a k‐mer length of 23 and a minimum k‐mer match length of 25. For museum specimens, sequencing reads from four libraries were merged after trimming to generate a single file for each individual. Trimmed and merged fastq files were then mapped to a high‐quality reference genome of the gull‐billed tern generated by the Vertebrate Genome Project (VGP, GenBank Accession Number: GCA_045787485.1) using bwa‐mem2 (v. 2.2.1, Vasimuddin et al. 2019). Accurate read pairing was verified using the *fixmate* option of samtools (v. 1.11, Danecek et al. 2021), followed by sorting, indexing, and conversion of the resulting SAM files into BAM format. Subsequently, samtools *markdup* was used to identify and remove duplicates. For museum samples, potential DNA damage due to cytosine deamination or conversion into uracil was assessed using MapDamage2 (Jónsson et al. 2013), and BAM files were rescaled, although only low levels of DNA damage were detected. Single nucleotide polymorphisms (SNPs) were identified using bcftools *mpileup* (v. 1.11, Danecek et al. 2021), with filters for a minimum base quality of 20, and a minimum mapping quality of 20, followed by SNP calling with bcftools *call*. The resulting VCF files were merged per scaffold and filters for a SNP quality > 20, read depth between the total number of samples and twice the average read depth, and mapping quality > 30 were applied. Sites with a read depth < 4 were set to “missing”, and individuals with less than half of the genome‐wide average read depth, as well as SNPs with > 20% missingness were excluded from our dataset. Since our species‐specific reference genome was a de novo assembled genome missing chromosomal annotations, we used a synteny‐based approach to assign chromosomal positions. Specifically, to organize scaffolds by chromosome and location, we used lastz (https://github.com/lastz/lastz.git) to align/map scaffolds to the available chromosome‐level reference genome of the related common tern ( *Sterna hirundo* , GenBank Accession Number: GCA_009819605.1). We then excluded SNPs on scaffolds that corresponded to the sex chromosomes and the mitogenome. To further assess the integrity of our museum samples and identify potential sequencing or preservation biases, we calculated the average sequencing depth per individual and examined the mutational spectrum using vcftools, comparing both metrics between museum and contemporary samples (Figure S1, Table S1).

### 2.3 Genome‐Wide Diversity

Given that relatedness and linkage disequilibrium (LD) can affect population structure (Anderson and Dunham 2008; Zou et al. 2010; Conomos et al. 2015), we assessed both using PLINK (v.1.90, Purcell et al. 2007) prior to running further analyses. Our relatedness analysis led to the exclusion of four individuals (two historic and two contemporary German), each removed as one of a closely related pair (parent‐offspring or sibling). LD pruning was performed with an *r* ^2^ threshold of 0.2, window size of 100 bp and step size of 50 bp. The filtered VCF file containing all samples was then split into two files: one including only historic and German samples and another retaining samples from all three contemporary populations (Germany, Italy, Spain). Both VCF files were filtered using PLINK, keeping biallelic autosomal SNPs with minor allele frequency of > 0.05, minimum allele count > 1, minimum allele frequency < 0.01 and < 10% missing data.

Genome‐wide nucleotide diversity (*π*) per 15 kb windows was calculated for each population using genomics_general (https://github.com/simonhmartin/genomics_general). We then calculated individual heterozygosity for each population using PLINK, and a Welch *t*‐test was conducted in RStudio (version 4.3.3; R Core Team 2022) to assess differences between populations. To assess whether population expansion or contraction occurred, Tajimas' *D* (Tajima 1989) was calculated in 15 kb windows using vcftools. Genome‐wide population structure was analysed using principal component analysis (PCA) performed in PLINK to identify clustering based on genetic similarities, and these and all other results were visualised using the “ggplot2” package (Wickham 2016). Pairwise *F*_ST_ and absolute divergence (*D*_*XY*_) were calculated as two independent measures of genetic differentiation between populations using genomics_general with 15 kb sliding‐windows, with *F*_ST_ reflecting current, and *D*_*XY*_ accounting for ancestral differences. To characterize ancestral population structure, admixture analyses were performed using ADMIXTURE (v. 1.3.0, Alexander et al. 2009) for eight ancestral populations (*K*), with each *K* run repeated 20 times using different seeds per *K* to assess convergence of runs within each *K*. ADMIXTURE was run on the museum/German dataset and the contemporary dataset separately. To determine the optimal *K*, we calculated the cross‐validation error for each *K* as a measure of accuracy. The *K* with the lowest error value, indicating the most accurate result, was considered to be most likely (Figure S2).

In addition, we ran GONE (Santiago et al. 2020) for each population to estimate the historic effective population size (*N*_e_) in the recent past (i.e., 250 generations). GONE estimates *N*_e_ by quantifying genome‐wide patterns of LD across different recombination distances and identifies the demographic history that explains the observed LD decay best. To run GONE, we used phased genotype data in PLINKs ped and map format. Because our gull‐billed tern genome assembly consisted of multiple scaffolds (c. 28,000) and GONE only supports a maximum of 200 chromosomes, we restricted this analysis to the largest scaffold (101,971,127 bp). To ensure sufficient SNP density within this single scaffold, we used a subset of both historical and contemporary German samples, with 10 samples from each population and a mean coverage > 17×. To account for the missing genetic distance between markers, we chose a recombination rate of 1.6 cM/Mb (centimorgans per Megabase) based on values of common terns (Malinovskaya et al. 2018) and ran 40 replicates for each population. Although the contemporary German population derived from the historic population, we analysed them as two separate populations to assess whether the inferred demographic histories converge to one shared ancestral *N*_e_ as would be expected under a population‐wide decline or whether the contemporary population shows a persistently lower *N*_e_, indicative of a founder effect in which the contemporary German population originated from a small subset of the historic population.

### 2.4 Runs of Homozygosity

To assess the level of inbreeding in the contemporary and historic populations, we assessed the genome‐wide runs of homozygosity (ROH). ROH are long, continuous homozygous regions in the genome, believed to have arisen from a common ancestor (Ceballos et al. 2018). Shorter ROH suggest distant inbreeding, while longer ROH indicate more recent inbreeding events (Curik et al. 2014). ROH were assessed without applying prior LD or minor allele frequency (MAF) filtering, following recommendations of Meyermans et al. (2020). For more robust estimates, and since PLINK tends to overestimate the inbreeding coefficients based on ROH (Silva et al. 2024), we calculated the individual realized inbreeding coefficients (*F*_ROH_) using the R package RZooRoH (v.0.4.1, Bertrand et al. 2019), with the historic and modern datasets analyzed separately. This R package uses a hidden Markov model to detect segments of homozygosity‐by‐descent (HBD; identified as ROH) and non‐HBD segments. Because the length of ROH is indicative for the timing of inbreeding events, the RZooROH package categorizes HBD segments lengths into approximate generation classes. A predefined “zoomodel” for 11 different HBD classes was implemented with *R*_k_ values of 4, 8, 16, 32, 64, 128, 256, 510, 1024, 2048 and 4096 for HBD segments, and > 4096 for non‐HBD segments. Each of these *R*_k_ values corresponds to approximately twice the number of generations since the inbreeding event occurred, thus representing inbreeding events occurring 2, 4, 8, 16, 32, 64, 128, 256, 510, 1024 and 2048 generations ago. A Welch *t*‐test was conducted in RStudio to assess differences in *F*_ROH_ between populations.

## 3 Results

The 27 contemporary gull‐billed tern samples had a mean read depth of 21× and a genotyping rate of 99.4%, while the 18 historic genomes exhibited a mean read depth of 18× with a genotyping rate of 99.0%. After filtering and LD pruning, the historic German dataset contained 1,540,408 SNPs, while the dataset including the genomes of all contemporary samples contained 1,263,070 SNPs.

Nucleotide diversity was similar across the three contemporary populations (Table 1), but the German population exhibited significantly lower observed heterozygosity values (*H*_O_) than the Spanish (*t* = −23.085, *p* ≤ 0.001) and Italian (*t* = −14.583, *p* ≤ 0.001) populations, although within the German population, these values were higher than the expected heterozygosity (*H*_E_) under Hardy–Weinberg equilibrium. A similar pattern of higher *H*_O_ than *H*_E_ was also found within both Mediterranean populations that did not differ significantly in their *H*_O_ values (*t* = −0.753, *p* = 0.473).

**TABLE 1:** Average genetic diversity parameters calculated in 15 kb windows for gull‐billed terns from three contemporary European populations and one historic population.

Population	Nucleotide diversity (*π*)	Mean observed heterozygosity (*H*_O_)	Mean expected heterozygosity (*H*_E_)	Tajimas' *D*
Germany (*n* = 15)	0.210	0.286	0.268	0.215
Italy (*n* = 6)	0.223	0.352	0.321	0.149
Spain (*n* = 6)	0.224	0.356	0.320	0.134
Historic (*n* = 18)	0.285	0.235	0.221	0.383

**FIGURE 2:**
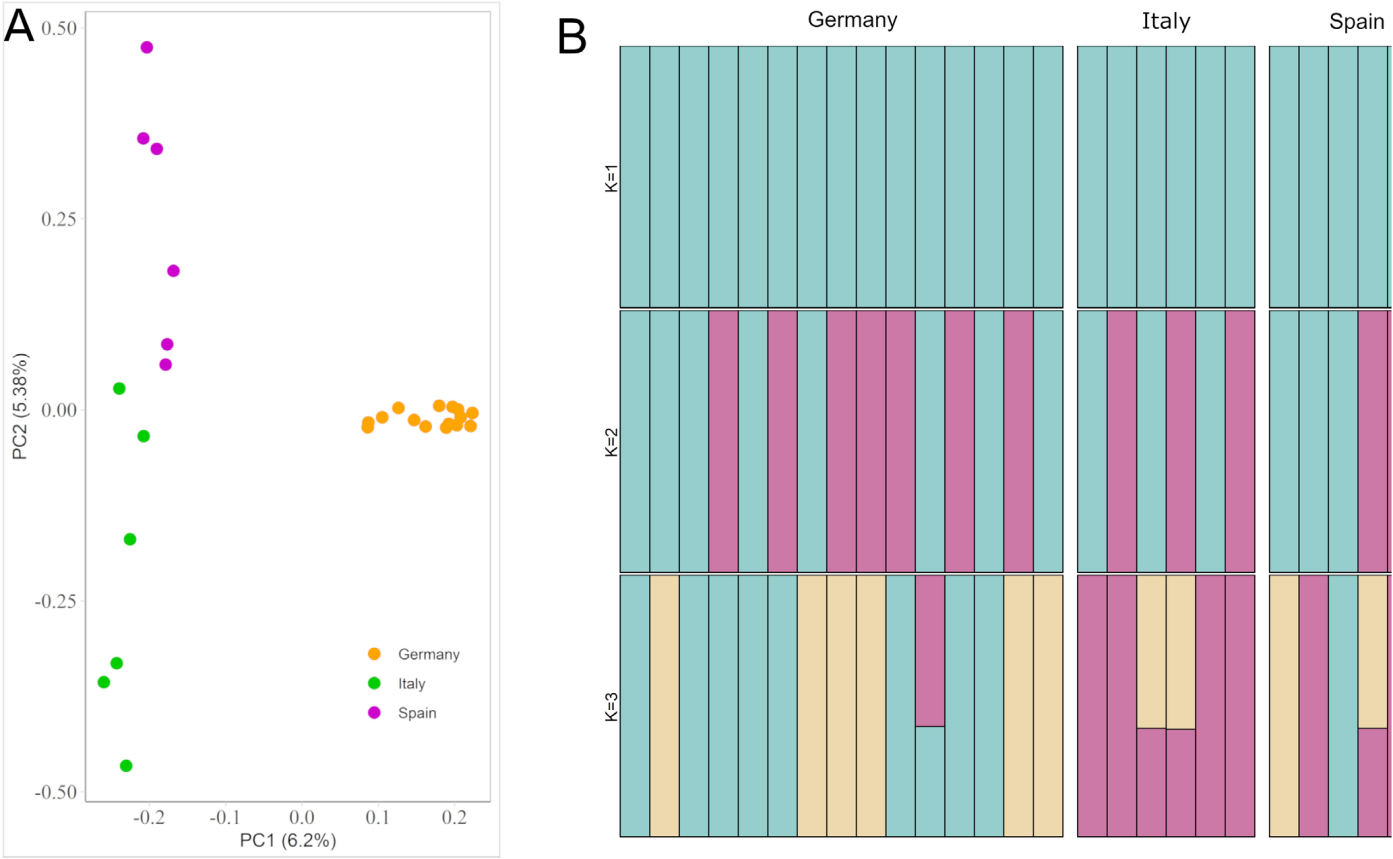
Population structure based on (A) PCA and (B) admixture analysis of the three contemporary European gull‐billed tern populations. In (A), individuals are grouped based on their genetic similarity and color coded per population, following the same scheme as in Figure 1A. Percentages are calculated by dividing the estimated eigenvalues for each principal component by their overall sum and multiplying by 100. In (B), admixture plots assuming 1 to 3 ancestral populations are shown. Each bar represents the ancestral proportions per sample (Germany *n* = 15, Italy *n* = 6, Spain *n* = 6).

Tajimas' *D* values were positive in all populations, but varied slightly, being highest in the contemporary German and lowest in the historic population (Table 1). The PCA for the contemporary populations revealed local clustering (Figure 2A). The first principal component (PC1) separated the German from the Mediterranean populations, while the second principal component (PC2) distinguished the two Mediterranean populations. Based on the cross‐validation error (Figure S2), the admixture analysis suggested the most likely scenario to be that the German and Mediterranean samples came from one single ancestral genetic cluster (*K* = 1, Figure 2B). In addition, mean pairwise *F*_ST_ values per window revealed low genetic differentiation between the populations, with the lowest *F*_ST_ between Spain and Italy (Table 2). The genetic distance (*D*_*XY*_) between populations was moderate and comparable among populations (Table 2).

**TABLE 2:** Pairwise *F*_ST_ (bold) and *D*_*XY*_ comparison (below the diagonal) and beeline distance (above the diagonal) for three contemporary European gull‐billed tern populations.

Population	Germany	Italy	Spain
Germany (*n* = 15)	—	968.58	2047.77
Italy (*n* = 6)	**0.008** / 0.220	—	1682.55
Spain (*n* = 6)	**0.007** / 0.220	**0.001** / 0.224	—

*Note:* The beeline distance (in km) was calculated in QGIS (QGIS Development Team 2022) using the “distance matrix” function.

**FIGURE 3:**
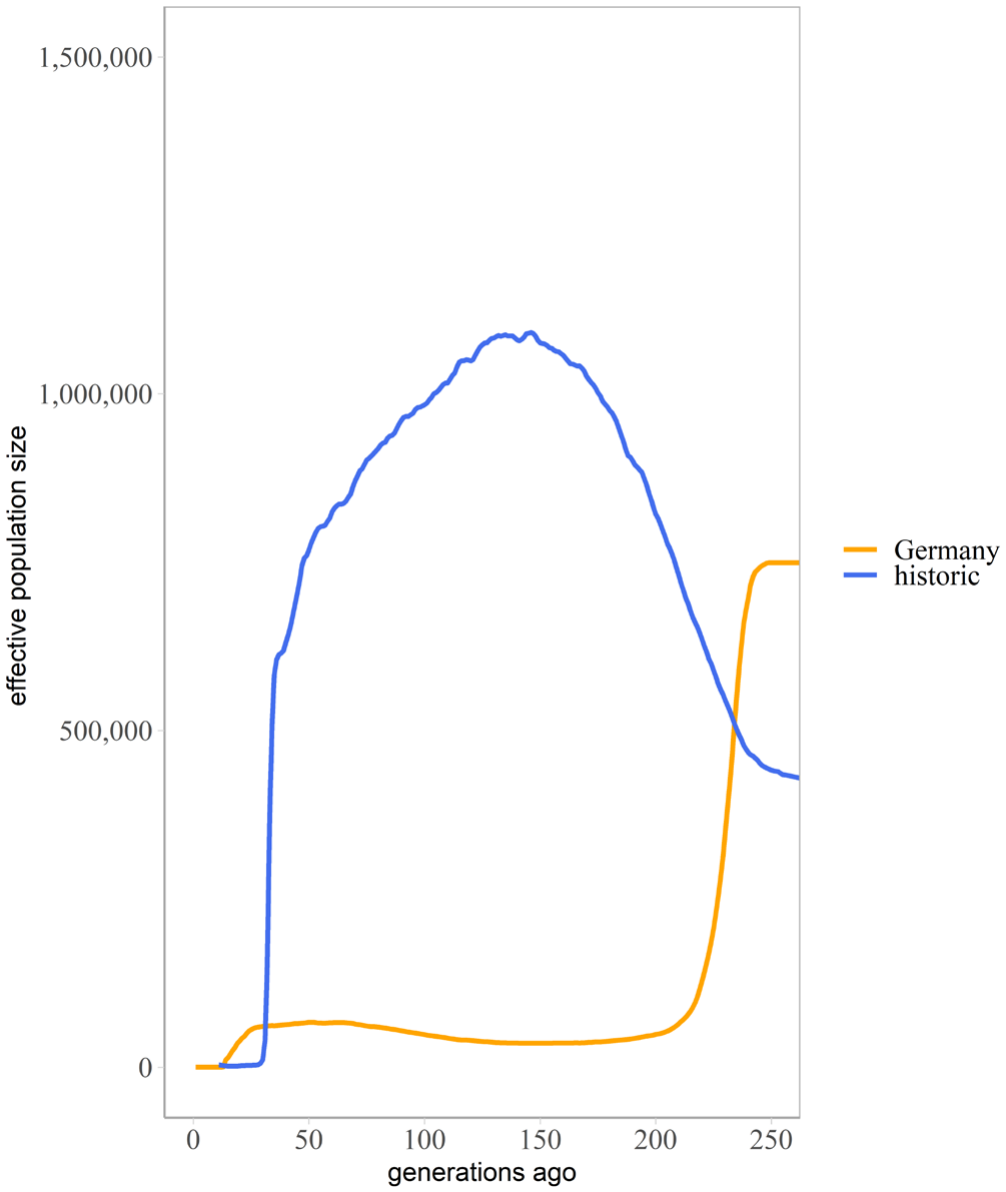
Effective population sizes estimated for the historic (blue, based on *n* = 10 individuals) and extant German (orange, based on *n* = 10 individuals) population of gull‐billed terns. Effective population size estimates for the extant population were consistently lower than those for the historic population, consistent with a founder effect in which the contemporary population originated from a restricted subset of the historic population. A first population decline occurred around 150 generations ago in the historic population, with a smaller second decline in the extant population approximately 30 generations ago. The historic samples indicate that the ancestral population was larger, but a population decline might have started earlier than seen in the monitoring data. The inferred *N*_e_ for the historic population was 2862, while the contemporary German population had a *N*_e_ of 250.

Focusing on the current German population and its ancestors, the PCA revealed little differentiation between them (Figure S3A). However, potential temporal clustering was observed on the first PC. The admixture analysis also identified one single ancestral cluster for the historic and contemporary German population (Figure S3B). Estimates of effective population size (*N*_e_) over the last 250 generations suggested a first population decline to have started in the historic population around 150 generations ago, with a smaller second one taking place in the extant population approximately 30 generations ago, resulting in a lower *N*_e_ in the contemporary population (Figure 3). Effective population size estimates for the contemporary population were consistently lower than those for the historic population, indicative of a founder effect in which the contemporary German population originated from a small subset of the historic population.

The length of ROH across all populations (both contemporary and historic) averaged 0.014 Mb. While most of the ROH lengths were shorter than 1 Mb (*n* = 4236), Spain had the longest ROH at 8.1 Mb, with a mean length of 0.014 Mb. The longest ROH in the historic samples was 1.8 Mb (mean of 0.012 Mb), while both the German population (mean 0.017 Mb) and the Italian population (mean 0.016 Mb) had runs up to 5.9 and 2.1 Mb, respectively (Figure S4). A total of 188 ROHs larger than 1 Mb were identified across all populations, with 11 of them exceeding 3 Mb (Table S1). Of these, eight ROHs were found in the German population, three in the Spanish, and none in the Italian and historic population.

**FIGURE 4:**
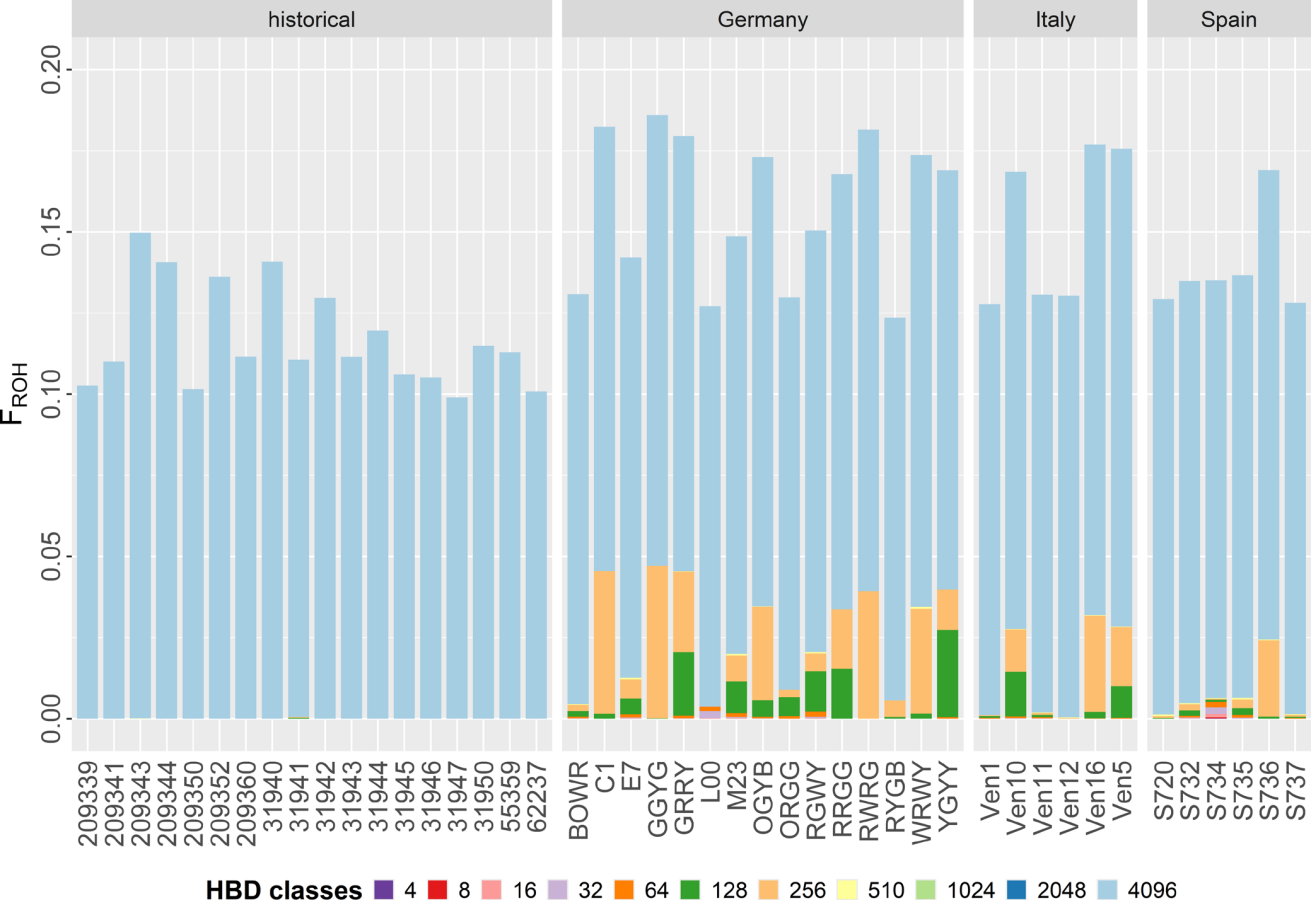
Genome‐wide estimates of individual inbreeding coefficients (F_ROH_) in three contemporary and one historic population of gull‐billed terns. Bars are divided by colors representing the proportion of the genome assigned to 11 different length classes of homozygosity‐by‐descent (HBD). HBD class 4 represents the longest, and HBD class 4096 the shortest segment. Each segment corresponds to an inbreeding event occurring approximately 2, 4, 8, 16, 32, 64, 128, 256, 510, 1024, and 2048 generations ago.

The realized inbreeding coefficient *F*_ROH_, based on the proportion of the genome covered by ROH, differed between the contemporary and historic populations (Figure 4). While the historic population revealed *F*_ROH_ values corresponding to a distant past (c. 2048 generations), all contemporary populations displayed overall higher *F*_ROH_ values and more recent inbreeding events. As such, *F*_ROH_ values were not significantly elevated in the German compared to both the Spanish (*p* = 0.105) and Italian populations (*p* = 0.256). However, in comparison to their ancestors, *F*_ROH_ values of the contemporary German population were significantly higher (*p* = 0.002). Similarly, the Spanish (*p* = 0.001) and Italian (*p* = 0.003) populations had significantly higher *F*_ROH_ values compared to the historic German population.

## 4 Discussion

We conducted the first genome‐wide assessment of genetic diversity in the last remaining, and endangered, Central European gull‐billed tern population (located in Germany), and compared it to that of two thriving European populations in Spain and Italy. Building up on a recent study based on mitochondrial DNA (mtDNA) (Schnelle, Rollins, et al. 2024), which indicated high genetic diversity and signs of population contraction, but also potential population connectivity, this whole‐genome analysis provided further support for the initial findings. To further characterize the impact of the recent population decline on the German population, we complemented our dataset with historic samples from birds from the ancestral, pre‐decline population of the contemporary German birds. The inclusion of these historic DNA samples (hDNA) similarly revealed that the last Central European population has maintained levels of genetic diversity, but does show an increase in inbreeding. Although the current inbreeding coefficient (*F*_ROH_) remains at a low level, and is similar to that of the other contemporary populations, further inbreeding could lead to inbreeding depression and become a concern in the future.

Consistent with Schnelle, Rollins, et al. (2024), we found that the genome‐wide nucleotide diversity of the endangered German population of gull‐billed terns is comparable to that of the two thriving Mediterranean populations. However, the German population exhibits significantly lower mean observed heterozygosity (*H*_O_) values compared to these two Mediterranean populations, indicative of overall lower genetic variability. Interestingly, the observed heterozygosity of all populations is higher than expected (*H*_E_) under Hardy–Weinberg equilibrium, indicating an excess of heterozygotes in all populations. Since our mutational spectrum analysis revealed very similar substitution patterns between the contemporary and historic samples (Figure S1), it is unlikely that biases from degraded hDNA influenced our heterozygosity estimates. One possible explanation for the observed patterns therefore could be the “isolate‐breaking” effect, where a recent reproducing immigrant causes an increase in heterozygotes (Wahlund 1928; Karamanlidis et al. 2018). If no further immigration occurs, this pattern would exist for several generations until a new genetic equilibrium is established (Hedrick et al. 2018). Although we did not directly assess gene flow or migration rates, the presence of the excess, together with the admixture results indicating a shared ancestral population (Figure 2B) and the low *F*_ST_ values between the contemporary populations (Table 2), is consistent with connectivity between the three populations that could be maintained by low levels of gene flow. The lowest *F*_ST_ was observed between the Spanish and Italian populations, which are also in close geographical proximity (Table 2). As the initial recorded nesting event in Italy in the late 1940s (Brandolini 1950) predates the breeding records for the Extremadura colony in Spain, dating back to the 1980s (Corbacho et al. 2009), the low genetic differentiation between those two populations may be due to ongoing immigration. Support for this comes from one individual that was ringed as a chick in Spain and recaptured as a breeding adult in Italy (personal communication), fitting with the general notion that juveniles tend to prospect and visit non‐natal breeding areas (Dittmann et al. 2007). As shown in other species, only a small number of migrants is needed to equalize allele frequencies across colonies (Mills and Allendorf 1996; Del Lama et al. 2002; Oomen et al. 2011; Davis et al. 2021).

Genetic differentiation between the two Mediterranean populations and the German population was slightly higher, although values still exceeded 0.05, which generally indicates low genetic differentiation (Wright 1965, 1978). At present, no immigration into the German population has been observed directly by reading the ring of a breeding bird that was ringed as a chick in another population, but there are unringed birds among the breeders, such that immigration cannot be excluded. Alternatively, an excess in heterozygotes can also arise from other processes unrelated to gene flow or migration. Heterozygous individuals, for example, could have a selective advantage, resulting in elevated heterozygosity (Krüger et al. 2001; Hedrick 2012). Furthermore, negative assortative mating for genetic make‐up can increase heterozygosity and maintain genetic diversity, resulting in the observed pattern (Workman 1964; Hedrick 1992; Hedrick et al. 2018).

Finally, low *F*_ST_ values can also arise from balancing selection maintaining similar allele frequencies across populations, reducing genetic differentiation. While *F*_ST_ values are rather quickly reduced by homogenizing allele frequencies between populations, absolute divergence values such as *D*_*XY*_ remain unaffected, as this measure reflects nucleotide differences that have accumulated since divergence (Cruickshank and Hahn 2014). Given that *D*_*XY*_ is affected by ancestral diversity or substitution rate, it is particularly elevated in regions where substitutions have been fixed in a population, for example, as part of positive selection (Chase et al. 2021). The combination of low *F*_ST_ and moderate *D*_*XY*_ observed in our study suggests that there are specific regions in the genome where the populations show more differentiation. These regions could represent retained differences, resulting in local adaptation and population structure, as further indicated by the clustering observed in the PCA. Thus, although *F*_ST_ is low and both the admixture results and the excess of heterozygosity may indicate gene flow, the presence of moderate *D*_*XY*_ and clustering in the PCA suggest that some degree of differentiation has started to occur.

The overall lower observed heterozygosity (*H*_O_) in the German population is likely due to the past population decline, reducing the breeding pair numbers from 500 to 52 over 80 years (Figure 1C). With fewer individuals and less genetic variation, this decline could have increased the potential for mating between more closely related individuals, resulting in longer ROH (Curik et al. 2014), homozygous deleterious mutations (Stoffel et al. 2021; Bertorelle et al. 2022) and elevated long‐term extinction risk (Crooks et al. 2017). If severe inbreeding occurs, species often exhibit several large ROH, as shown for example in the California condor (*Gymnogyps californanus*) where ROH extended up to 16.8 Mb (Robinson et al. 2021). While most observed ROH in our study were smaller than 3 Mb, a size also common in outbred species (Ceballos et al. 2018), long ROHs (3–8 Mb) were only detected in the German and Spanish population. The majority of these long ROHs was found in the German population, although the longest ROH was observed in the Spanish population, probably originating from an individual common ancestor eight generations (c. 78 years) ago. In addition, the significantly higher inbreeding coefficient *F*_ROH_ in the German population compared to the ancestral population, as well as the overall lower *H*_O_ in the German population, indicate that this population has been impacted by the population decline. This supports the idea that some regions in the genome show more differentiation as a result of reduced variability due to the population decline. However, the overall low levels of *F*_ROH_ observed in the contemporary populations, despite being significantly higher than in the historic population, suggest that neither of these populations have experienced severe inbreeding or long‐term isolation yet. The long generation time of gull‐billed terns could have mitigated these effects, as observed in other long‐lived species (Hailer et al. 2006). Population genetic theory predicts that genetic diversity loss is slow, often taking several hundred generations (Amos and Balmford 2001). With a generation time of 9.75 years (Bird et al. 2020), it would take approximately 57 generations (over 550 years) for the German population to lose 25% of its heterozygosity, when reduced to an effective *N*_e_ of 100 (Frankham et al. 2017). As the population decline only started about 80 years ago, it is unlikely that it has caused the reduced heterozygosity and elevated inbreeding coefficient. This suggests that lower heterozygosity levels may have already been present prior to the decline. Interestingly, we found that the observed heterozygosity was comparable between the contemporary German population and its ancestral counterpart, supporting that the recent population decline did not affect their heterozygosity. The ancestral population already showed higher *H*_O_ than *H*_E_, suggesting that this population had either engaged in negative assortative mating or had already been in contact with other populations, possibly through similar migrations routes (Sánchez et al. 2004) or gene flow from the former Alpine population (Berndt 2018). Given that the Alpine population was eradicated in the 1930s, some breeding individuals could have migrated further north to the Danish population, enhancing heterozygosity and genetic diversity in this population. Although we did not sample specimens that used to be part of the Alpine population, the admixture analysis between the historic and contemporary German population supported the existence of one ancestral panmictic population (Figure S3). The short ROH segments in the historic dataset support this, as potential population expansion could have reduced ROH length, as shown in other bird populations (Martin et al. 2023). However, DNA damage in the historic samples could have influenced ROH detection, as potential deamination patterns may have disrupted the runs (van der Valk et al. 2019; Walsh et al. 2024). This could also contribute to the lower *F*_ROH_ observed in the historic samples. Although we have taken additional steps to mitigate this and the mutational spectrum does not indicate a difference between the contemporary and historic datasets, we cannot fully exclude the possibility of DNA damage influencing the observed patterns. Given that we found ROH lengths of up to 5 Mb in the historical samples, however, we still believe that our results capture a genuine increase in inbreeding over time.

Our results suggest that the population decline in the former Danish population started earlier than previously thought. The *N*_e_ estimation indicated that the decline began approximately 60 generations ago, predating the observed recent decline. Our historic samples, collected between 1900 and 1928, represent a time when the Danish population consisted of about 250 breeding pairs (Møller 1975a). The Danish census population began to decline starting in 1949 (Møller 1975a), resulting in the consistently lower *N*_e_ found in the contemporary German population, indicative of a founder effect in which this population originated from a limited subset of the former Danish population. This suggests that the reduction in population size occurred gradually, with a pronounced bottleneck in the former Danish population approximately 60 generations prior to sampling. The observed decline in the census population that occurred approximately 8–9 generations ago further reduced the *N*_e_. The final *N*_e_ estimate in the German population (250.1) is above the threshold of 100, as recommended by Frankham et al. (2014) to avoid short term inbreeding depression. However, it remains below the suggested long‐term limit of 1000, highlighting the need for further studies that should focus on identifying potential migrants and migration routes to determine whether the suggested pattern of gene flow persists, or whether interactions with other factors, such as food availability (Schnelle, Winter, et al. 2024) or contamination (e.g., Schnelle et al. 2025) are posing threats to the German population.

Overall, our results suggest that although the three studied contemporary German, Italian, and Spanish populations of gull‐billed terns are not strongly genetically differentiated and retain high genetic diversity, they have experienced distinct population histories. The German population, in particular, has experienced two bottlenecks, resulting in an increased *F*_ROH_, higher mean ROH length, lower heterozygosity, and reduced effective population size in recent times. While ringing data does not confirm potential gene flow, the low genetic differentiation and excess of heterozygotes support the results of a previous mtDNA study (Schnelle, Rollins, et al. 2024) and suggest that the German population is not yet acutely endangered due to loss of genetic variability. The historic samples reveal that the German population has not been more genetically diverse in the recent past and has maintained genetic diversity after the second bottleneck. However, the significantly increased inbreeding coefficients, while currently still at a low level, may become a concern if gene flow is insufficient or isolation increases. As such, continued monitoring and protection of the population is crucial to assess population trends, identify potential immigration from other European populations, and ensure long‐term viability.

## Funding

This work was supported by the German Ornithologists' Society's Honig‐Förderung.

## Conflicts of Interest

The authors declare no conflicts of interest.

## Supporting information


**Data S1:** eva70192‐sup‐0001‐AppendixS1.docx.

## Data Availability

Whole‐genome sequencing data produced during this project are available at the European Nucleotide Archive under project ID PRJEB90146.
