# Supplementary material for: Using Historic and Contemporary Genomes to Assess the Genetic Consequences of a Population Decline in an Endangered Tern Population
Source: Evol Appl. 2026 Jan 2;19(1):e70192. doi: 10.1111/eva.70192 (PMC12759046; doi:10.1111/eva.70192)
Supplement: Supplementary file 1 — Data S1: eva70192‐sup‐0001‐AppendixS1.docx. [file EVA-19-e70192-s001.docx]

**Supplementary Material**

**to**

**Using historic and contemporary genomes to assess the genetic consequences of a population decline in an endangered tern population**

**Supplementary Tables**

Table S1. Comparison of statistics between historic and contemporary samples.

| **sample  age** | average depth (X) | number of heterozygous sites | number of transitions | number of transversions | number of missing sites |
| --- | --- | --- | --- | --- | --- |
| historic | 17.539 | 25,61,516 | 26,03,310 | 11,47,738 | 10,04,938 |
| contemporary | 20.796 | 24,83,493 | 26,19,333 | 11,36,536 | 567,392 |

**Supplementary Figures**


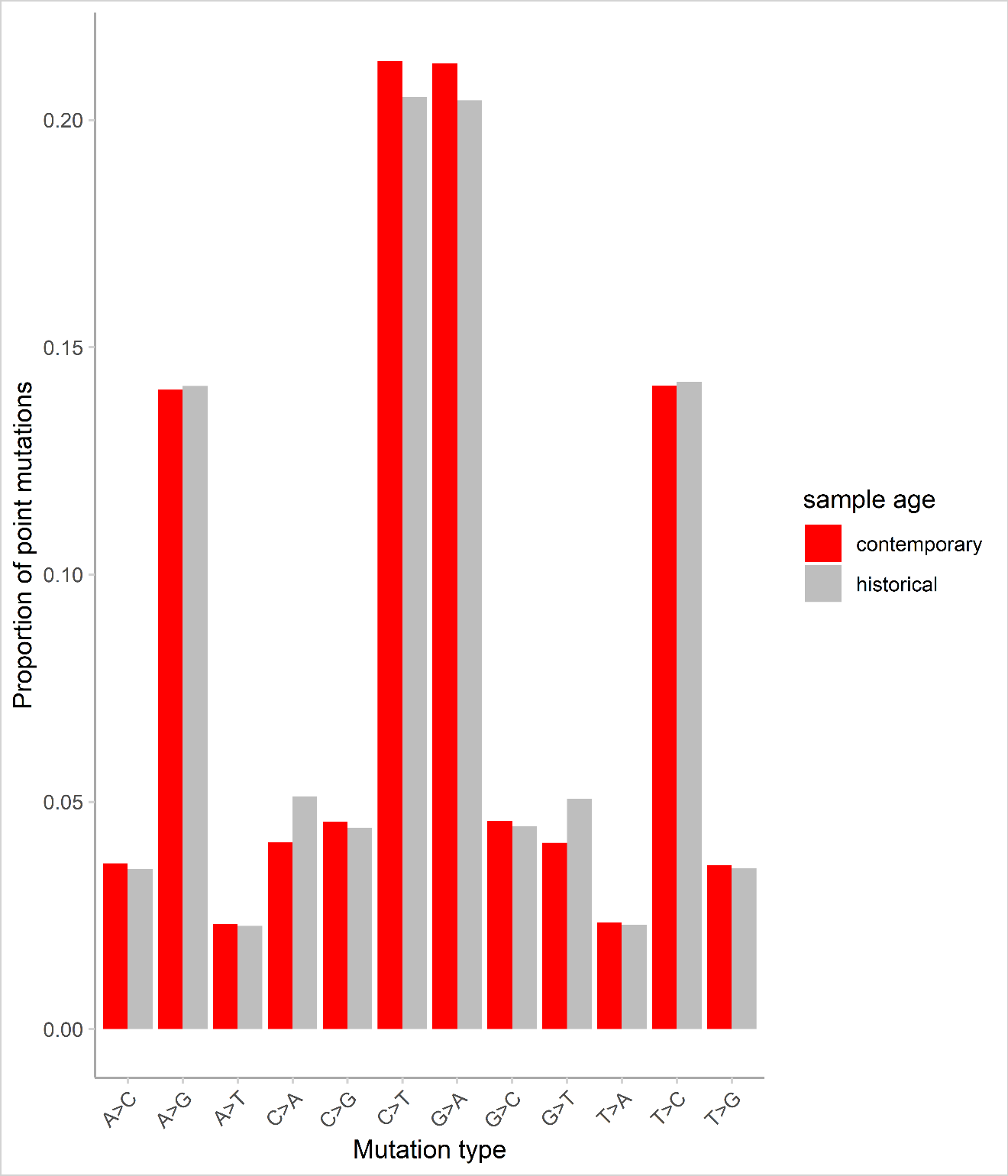


Figure S1. Mutational spectrum of the contemporary and historical DNA datasets, showing that mutation types did not differ significantly between historic and contemporary samples.

**
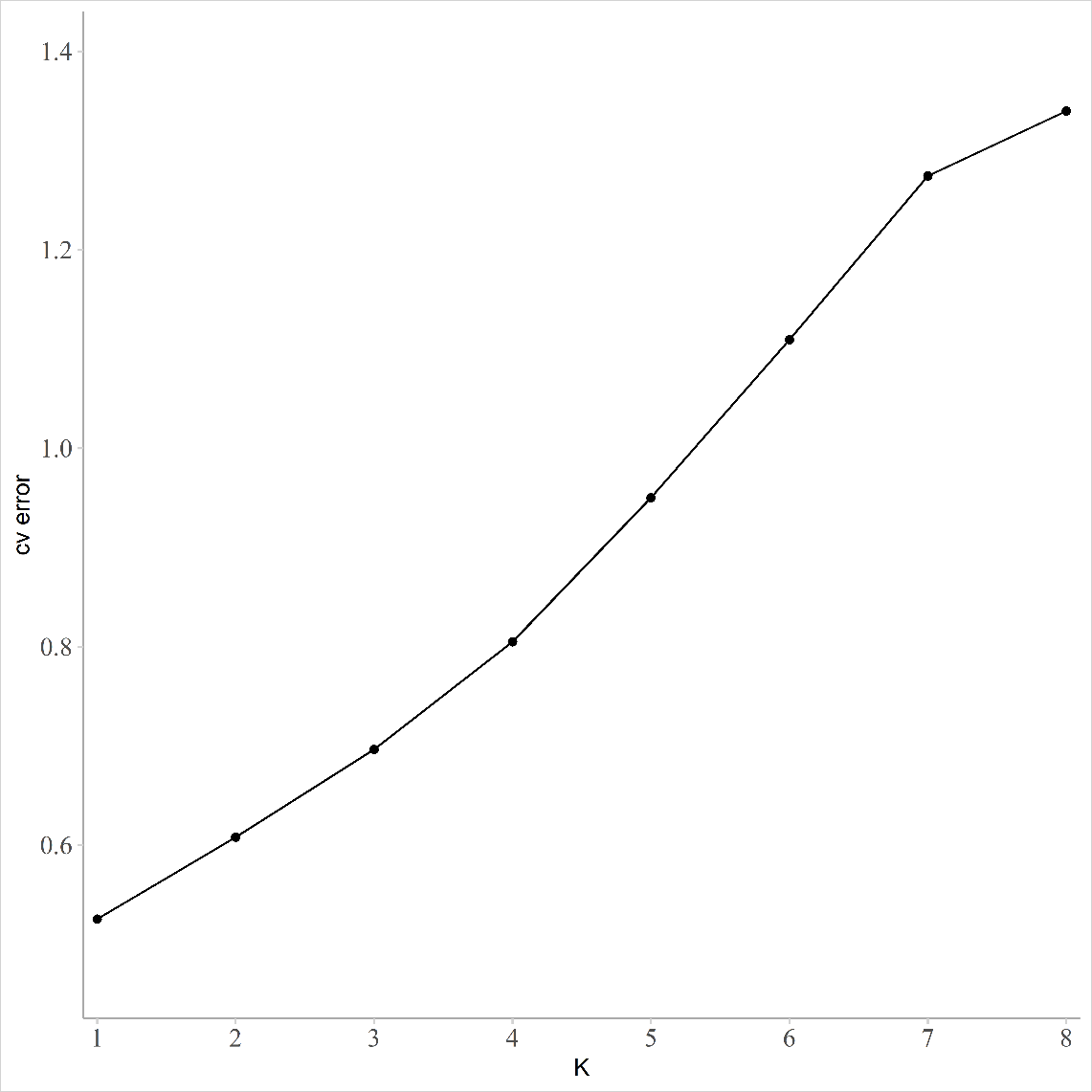
**

Figure S2. Cross validation (cv) error calculated for the Admixture analysis from K=1 to K=8. K=1 was chosen as the value that minimizes cross validation error and therefore resembles the most probable ancestral cluster.


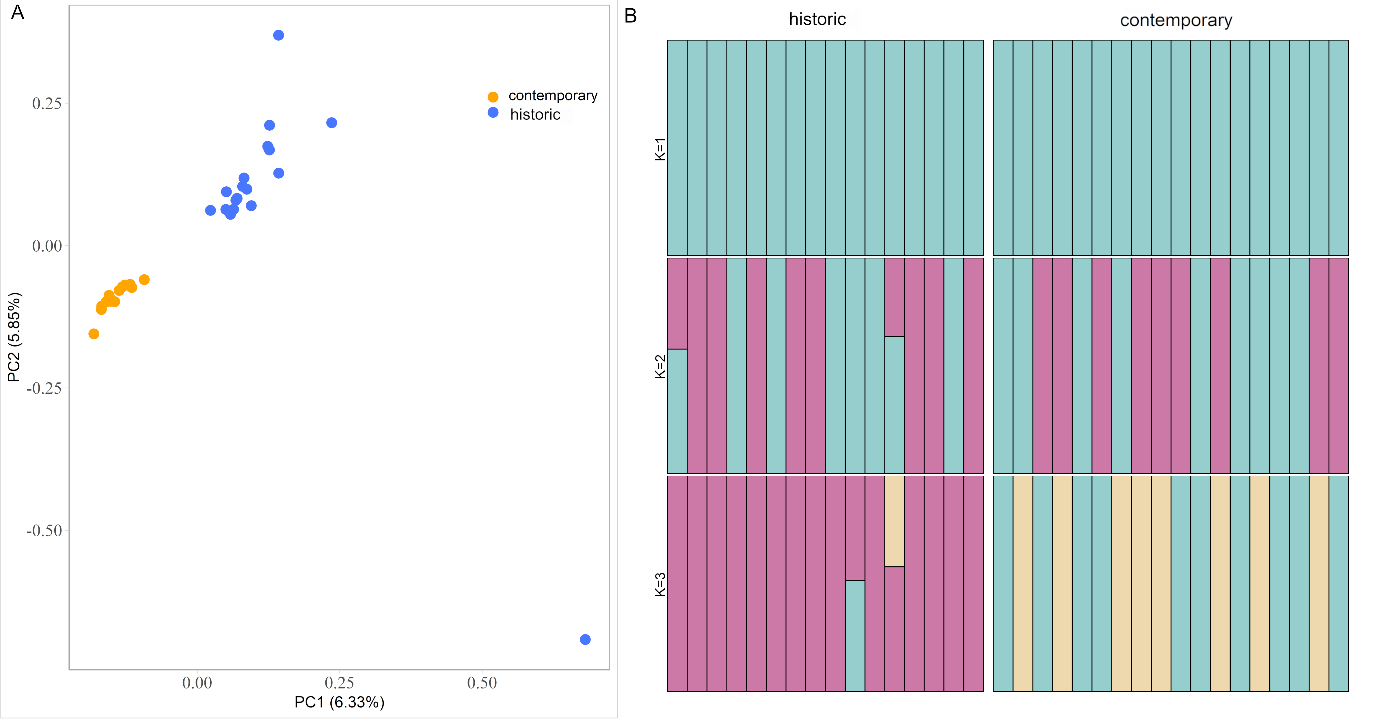


Figure S3. Population structure based on (A) PCA, and (B) Admixture analysis of the historic (n=18) and contemporary German gull-billed tern population (n=15). In (A), individuals are grouped based on their genetic similarity and colour coded per population (contemporary in orange, historic in blue). Percentages are calculated by dividing the estimated eigenvalues for each principal component by their overall sum and multiplying by 100. In (B), admixture plots assuming 1 to 3 ancestral population are shown. Each bar represents the ancestral proportions per sample.

**
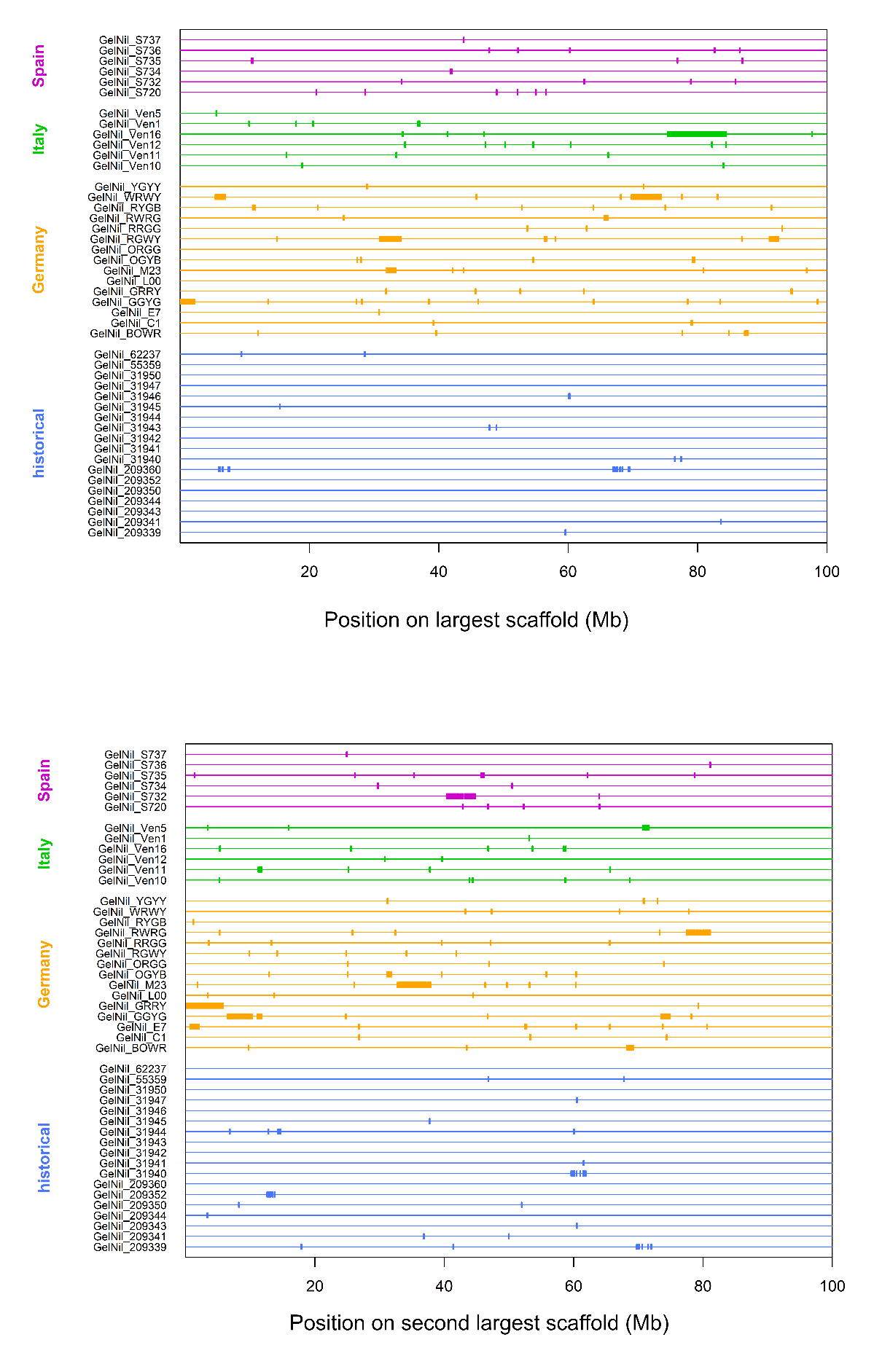
**

Figure S4. Runs of homozygosity (ROH) overlap plot on the two largest scaffolds for all four analysed populations of gull-billed terns.
